# Livin expression promotes keratinocyte release of inflammatory mediators in psoriasis

**DOI:** 10.1111/srt.13603

**Published:** 2024-02-08

**Authors:** Delu Che, Yazhuo Li, Bing Hang, Kaili Li, Kaijie Wang, Hao Wang

**Affiliations:** ^1^ Department of Dermatology Xi'an Jiaotong University Second Affiliated Hospital (Xibei Hospital) Xi'an China; ^2^ Center for Dermatology Disease Precision Medical Institute Xi'an China; ^3^ Department of Dermatology the 1st affiliated hospital, Jiangxi Medical College Nanchang University Nanchang China

**Keywords:** inflammation, keratinocytes, Livin, psoriasis

## Abstract

**Background:**

Psoriasis is a prevalent, long‐term skin condition characterized by inflammation. Keratinocytes (KCs) are important effector cells that release inflammatory factors and chemokines to promote the inflammatory cascade in psoriasis. However, the mechanisms underlying the activation of KCs in psoriasis remain unclear. Livin suppresses apoptotic proteins and directly affects the growth and spread of cancer cells. Livin expression reportedly increases significantly in lesions of patients with psoriasis; however, its specific role in KC activation remains unknown. This study aimed to examine the impact of Livin on KC activation and the subsequent release of inflammatory mediators.

**Methods:**

Immunofluorescence staining, reverse transcription‐quantitative polymerase chain reaction, enzyme‐linked immunosorbent assay (ELISA), and western blotting were used to assess Livin expression in patients with psoriasis, an imiquimod (IMQ)‐induced psoriasis‐like mouse model, and M5‐treated HaCaT cells. To investigate the role of Livin in KCs, we performed RNA sequencing and proteomic analysis of Livin‐knockdown (knockdown‐HaCaT) and negative control (NC‐HaCaT) cells. Gene Ontology and Kyoto Encyclopedia of Genes and Genomes were used for enrichment analyses. Moreover, the effect of Livin expression on the release of inflammatory mediators in KCs was verified using ELISA.

**Results:**

Livin expression was higher in KCs of patients with psoriasis than in those healthy controls. Livin expression in HaCaT cells treated with M5 increased significantly over time. Livin expression was higher in the skin lesions of the IMQ mouse model than in the control group. Proteomic analysis and RNA sequencing used to investigate the function of Livin in HaCaT cells revealed its potential role in mediating KC activation and inflammatory mediator release, which affected the pathology of psoriasis.

**Conclusions:**

Livin expression played an effect on KCs activation, which induced release of inflammatory mediators and up‐regulation of keratin. This study provides a new effector molecule for the mechanism of inflammatory response in psoriasis.

## 1 INTRODUCTION

Psoriasis is a prevalent and persistent inflammatory skin condition that recurs frequently, making it a significant concern in dermatology. Psoriasis is closely associated with hereditary diseases, infections, immune disorders, and other factors. The immune‐inflammatory cascade is a key factor in psoriasis progression. Although the complex interplay between immune cells and inflammatory factors^1^ regulates the inflammatory cascade, the complete mechanism remains unclear. Earlier research on the immune mechanisms of psoriasis primarily focused on the stimulation of T and dendritic cells. However, these studies have not elucidated the complete development of the initial inflammatory reaction in psoriasis.

Keratinocytes (KCs), also known as epidermal cells, play a crucial role in initiating, maintaining, and controlling skin immune responses. They can respond quickly and non‐specifically to antigens and external stimuli.^2^ The abnormal proliferation of KCs is a typical pathological feature of psoriasis. Previous studies have revealed the dual roles of KCs as initiators and blockers. KCs can cause histological alterations and engage with other immune cells to improve inflammation during early and advanced phases of psoriasis.^3^ During the initial phase of psoriasis, KCs attract and stimulate inflammatory cells, including dendritic cells, neutrophils, and macrophages.^4^, ^5^ Nevertheless, the precise process of KC stimulation during the initial phase of psoriasis remains unknown, and scientific investigations exploring the activation mechanisms and targets of KC are lacking.

The amino terminal of the Livin protein is composed of 1−3 baculovirus inhibitors of apoptosis protein (IAP) and baculovirus IAP repeat (BIR) domain, which have comparable lengths.^6^ Livin is a target for malignant tumor treatment because of its high expression in tumor tissues. Livin inhibits apoptosis mediated by mitochondria, death receptors, and chemotherapeutic agents. The primary mechanism by which it prevents cell death is the inhibition of caspase activity and the activation of the signaling pathway involving mitogen‐activated protein kinases 7 and 8.^7^ In addition, Livin expression was significantly upregulated in the KCs of patients with psoriasis. In 2018, our research group found that the expression of Livin in the KCs of skin lesions in psoriasis patients was higher than the healthy skin samples in JEADV, while the function of Livin in KCs and psoriasis was still unclear.^8^ Livin upregulation promoted KC proliferation and inhibited their apoptosis.^9^ However, the regulatory mechanisms of KC activation and the release of inflammatory mediators in psoriasis have not yet been reported. This study examined whether Livin affects KC activation in psoriasis and influences inflammation by regulating the expression of inflammatory mediators.

## 2 MATERIALS AND METHODS

### 2.1 Reagents

Vaseline was from Macklin Inc. (Shanghai, China), and imiquimod (IMQ) cream was bought from Inova Pharmaceuticals (3M Health Care, Leicestershire, UK). Recombinant human cytokine TNF‐α, IL‐17A, IL‐1A, IL‐22, and Oncostatin M were form Proteintech Group, Inc. (Chicago, Illinois, USA).

### 2.2 Human skin samples

Human skin samples were conducted in strict accordance with the guidelines set by our institution and with the approval of the Ethics Committee of Xi'an Jiaotong University (2022‐1040). Psoriasis vulgaris (case group) and healthy skin samples (control group) were obtained from the Department of Dermatology, Xi'an Jiaotong University Second Affiliated Hospital (*n* = 5). And the collection of written, informed consent from donors was obtained. Each tissue is divided into three parts, one was used for paraffin section, one was used for extracting the total proteins, and another one was used for extracting total RNA.

### 2.3 Livin expression analysis in human skin samples

The skin samples were regularly treated with a 4% solution of formaldehyde and then placed in paraffin for preservation. The paraffin sections were stained using hematoxylin and eosin (H&E) and immunofluorescent staining was performed using an anti‐Livin antibody (Abcam, ab97350).

Total proteins were extracted from the skin samples under cold conditions by RIPA lysis buffer, which included 10% phosphatase inhibitor and protease inhibitor (Roche Diagnostics). Livin protein expression was analyzed using a human Livin enzyme‐linked immunosorbent assay (ELISA) kit (Meilian Biotechnology Co., Ltd., Shanghai, China). Livin mRNA expression in human skin samples was analyzed by reverse transcription‐quantitative polymerase chain reaction (RT‐qPCR) after extraction of total RNA using TRIzol. The Livin primers were 5′‐GTCAGTTCCTGCTCCGGTCAA‐3′(forward) and 5′‐GGGCACTTTCAGACTGGACCTC‐3′(reverse).

### 2.4 Cell lines

Livin knockdown expression (Knockdown‐HaCaT) and negative control (NC‐HaCaT) cells were constructed by HIV‐1‐based lentiviral vectors with anti‐ puromycin gene (GenePharm, Shanghai, China). 1×10^5^ per well HaCaT cells were cultured in a 24‐well dish at 37°C with 5% CO_2_ for 24 h. Then the 2 mL RPMI 1640 medium, which contain 6 μg/mL polybrene and 1 mg/μL HIV‐1‐based lentiviral vectors, while without fetal bovine serum (FBS) was replaced the original medium and cultured the cells for 24 h, then replaced the media by fresh media. The cells were cultured for 72 h, the RPMI 1640 medium with 0.5 μg/mL puromycin was used to screen cells. And the livin expression level was assessed by RT‐qPCR, ELISA, and immunofluorescent staining. The HaCaT cells, knockdown‐HaCaT and NC‐HaCaT cells were grown using RPMI 1640 medium supplemented with 10% fetal bovine serum and 1:100 penicillin‐streptomycin.

### 2.5 IMQ‐induced psoriasis mouse model

C57BL/6 mice at the age of 10 weeks were acquired from Xi'an Jiaotong University's Experimental Animal Center. The IMQ‐induced psoriasis model was prepared by topically applying 5 mg of Aldara cream (containing 5% IMQ, INova Pharmaceuticals) or control cream on mouse skin for five consecutive days. The shaved dorsal areas of mice were subjected to daily application of IMQ cream. The visual and skin‐related characteristics of the lesions were documented (*n* = 9). Mice were euthanized by inhaling CO_2_, and their skin tissues were collected for analysis.

### 2.6 Histology

Samples of mouse skin were treated with 4% paraformaldehyde and then enclosed in paraffin. The paraffin sections were stained using H&E. Sections were dried at a temperature of 37°C for a duration of 30 min. Following this, they were pre‐incubated in a blocking solution consisting of normal goat serum (10%, v/v) and Triton X‐100 (0.2%, v/v) in PBS (pH = 7.4) for h at 25°C. After the pre‐incubation, the sections were incubated with an anti‐Livin antibody from Thermo Fisher Scientific (Shanghai, China) for 45 min. Subsequently, the sections were washed three times with PBS and Fluoro‐mount G (Southern Biotech) was added.

### 2.7 RNA‐seq and proteomic analysis

TRIzol method was used to collect the total RNA from Knockdown‐HaCaT and NC‐HaCaT cells. RNA‐seq analysis of transcriptomics was conducted by Berry Genomics Corporation located in Beijing, China. DESeq2 was the statistical power of this experimental design, which was calculated in NCBI BioProject PRJNA856067. RIPA lysis buffer was used to collect the total protein from Knockdown‐HaCaT and NC‐HaCaT cells. GeneChem Co., Ltd. (Shanghai, China) completed the proteomic analysis. The statistical strength of this experimental setup, computed in ProteomeXchange (PXD035174). R (https://www.r‐project.org/) was used to perform the analysis of sequencing data.

### 2.8 HaCaT cell activation and inflammatory mediators release analysis

NC‐HaCaT cells and knockdown‐HaCaT and were cultured in a 6‐well dish at 37°C with 5% CO_2_ for 1 night. Each well was supplemented with M5 (10 ng/mL of IL‐1A, IL‐22, IL‐17A, TNF‐α, and Oncostatin M), which was prepared using RPMI 1640 medium and incubated for 48 h. The negative control consisted of only RPMI 1640 medium. And the medium was collected for analysis MMP‐7, Cathepsin B, CXCL16, S100A8, S100A7, and S100A11 by ELISA Kits (Milian Biotechnology Co., Ltd., Shanghai, China).

## 3 RESULTS

### 3.1 Livin were upregulated in KCs of psoriasis

**FIGURE 1:**
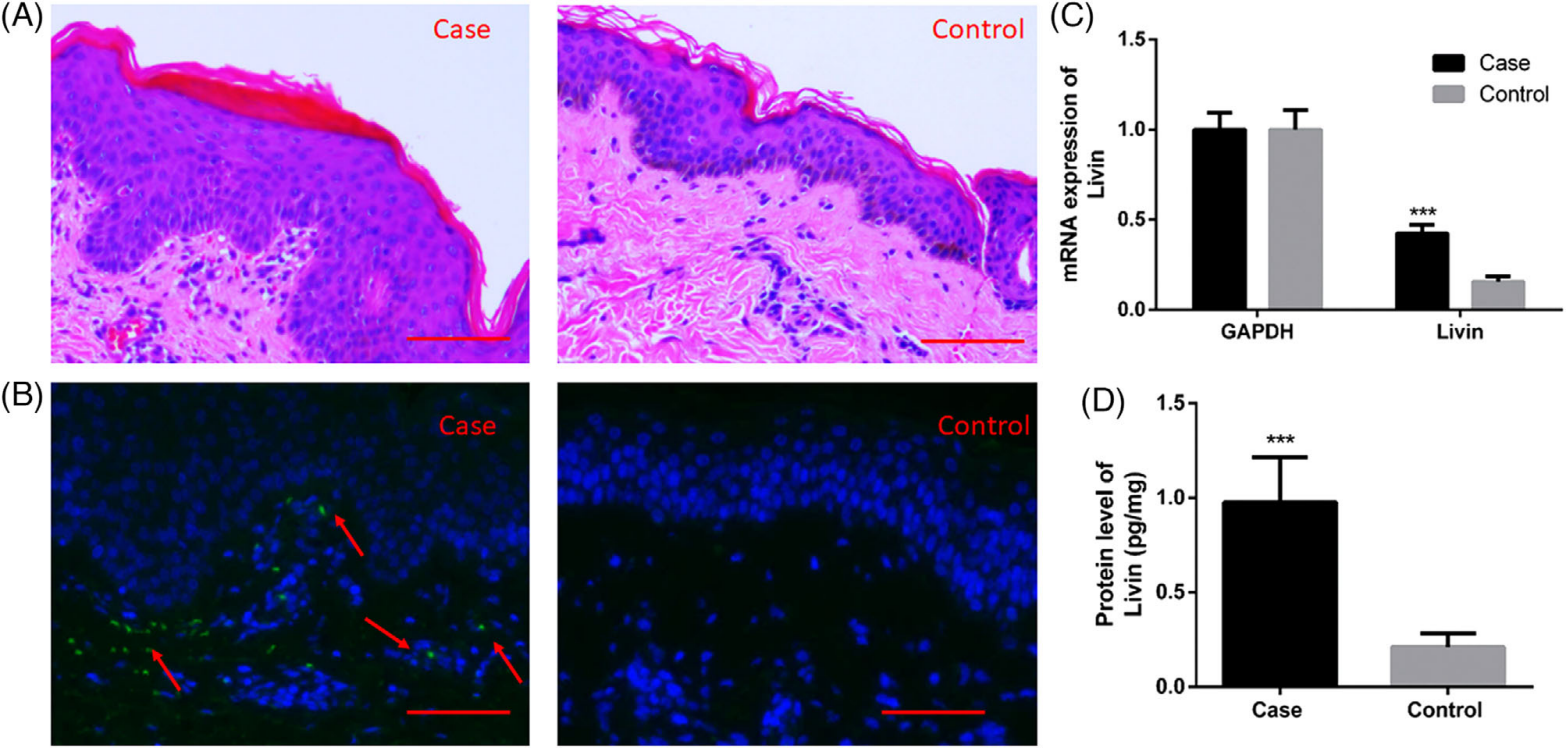
Livin expression was up‐regulated in KCs of psoriasis patients. (A) Pathological section of psoriasis patients (Case) and healthy samples (Control). (B) Livin was up‐regulated in KCs in case group than control group analysis by immunofluorescent staining. (C–D) The RNA level and protein level were increase in case group analysis by RT‐qPCR and ELISA. (*n* = 5, data are expressed as mean ± standard error of the mean (SEM) and analyzed one‐tail paired Student's *t*‐test. Differences were considered significant at ^***^ *p* < 0.001). ELISA, enzyme‐linked immunosorbent assay; KCs, keratinocytes; RT‐qPCR, reverse transcription‐quantitative polymerase chain reaction.

Immunofluorescence staining showed that compared with healthy control, patients with psoriasis had upregulated Livin expression levels in the KCs (Figure 1A,B). The total RNA and protein levels were further examined using RT‐qPCR and ELISA. Livin mRNA and protein levels were significantly elevated in patients with psoriasis (Figure 1C,D).

**FIGURE 2:**
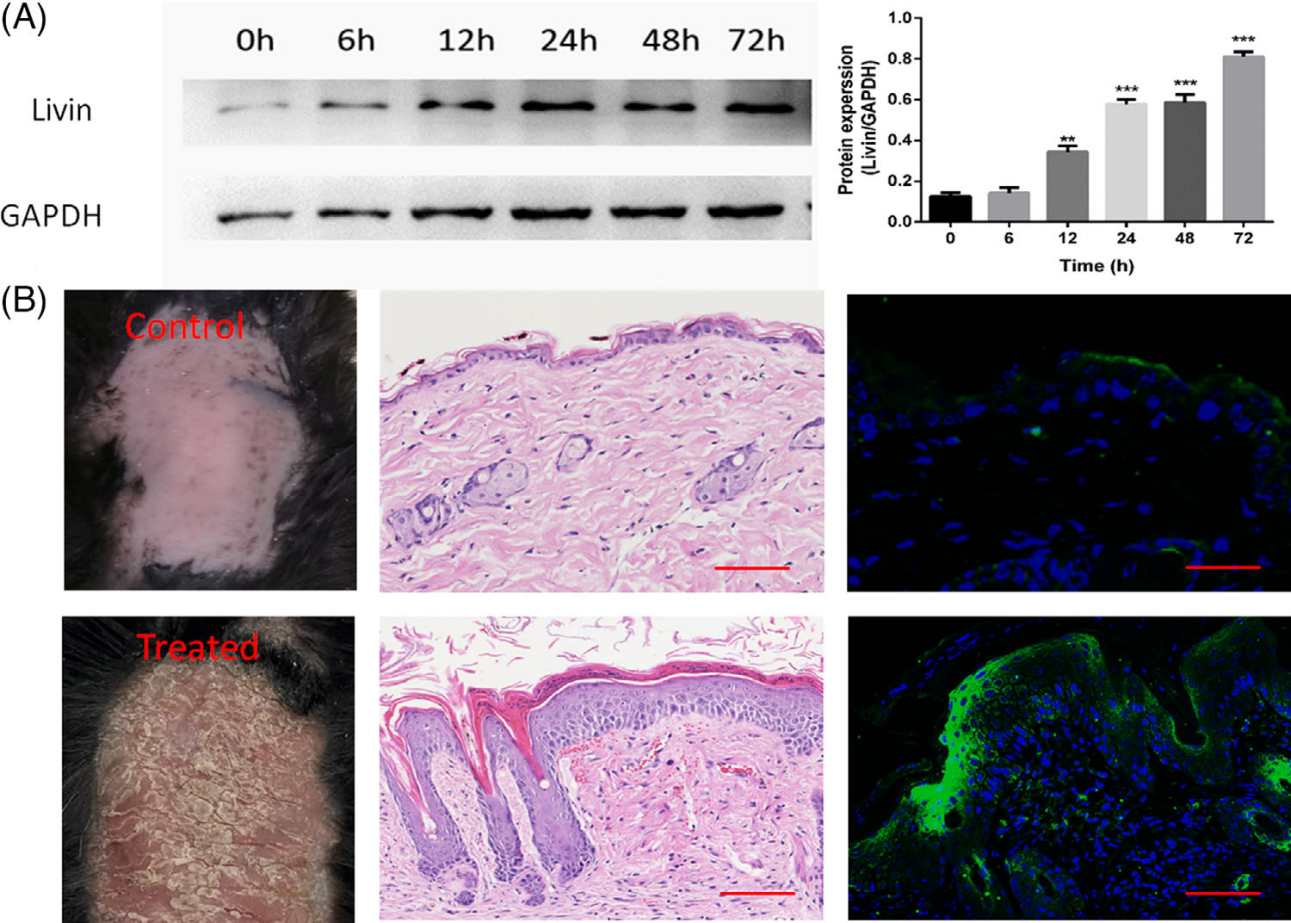
Livin expression was up‐regulated in M5‐Treated HaCaT cells and KCs of IMQ mouse model. (A) Livin was upregulated with time gradient after M5 treatment analysis by western blotting (data are expressed as mean ± SEM and analyzed one‐tail paired Student's *t*‐test. Differences were considered significant at ^**^ *p* < 0.01, ^***^ *p* < 0.001). (B) Livin was up‐regulated in KCs in IMQ treated group than control group analysis by immunofluorescent staining (*n* = 5). IMQ, imiquimod; KCs, keratinocytes.

Livin expression was analyzed in M5 (interleukin [IL]−1A, IL‐22, IL‐17A, tumor necrosis factor [TNF]‐α, and oncostatin M)‐treated HaCaT cells in a time‐dependent manner. The results of western blotting and ELISA showed that the Livin expression levels were significantly increased for 12 h after M5 treatment (Figure 2A). Similar results were obtained in the IMQ mouse model, where the Livin expression levels were higher in the IMQ‐treated KCs of skin lesions than in the control group, as determined using immunofluorescence staining (Figure 2B).

### 3.2 Functional analyses of Livin

To date, no study has analyzed the function of Livin in KCs. Here, we generated knockdown HaCaT and NC‐HaCaT cells and determined their Livin expression levels using RT‐qPCR, ELISA, and immunofluorescence staining (Figure S1).

**FIGURE 3:**
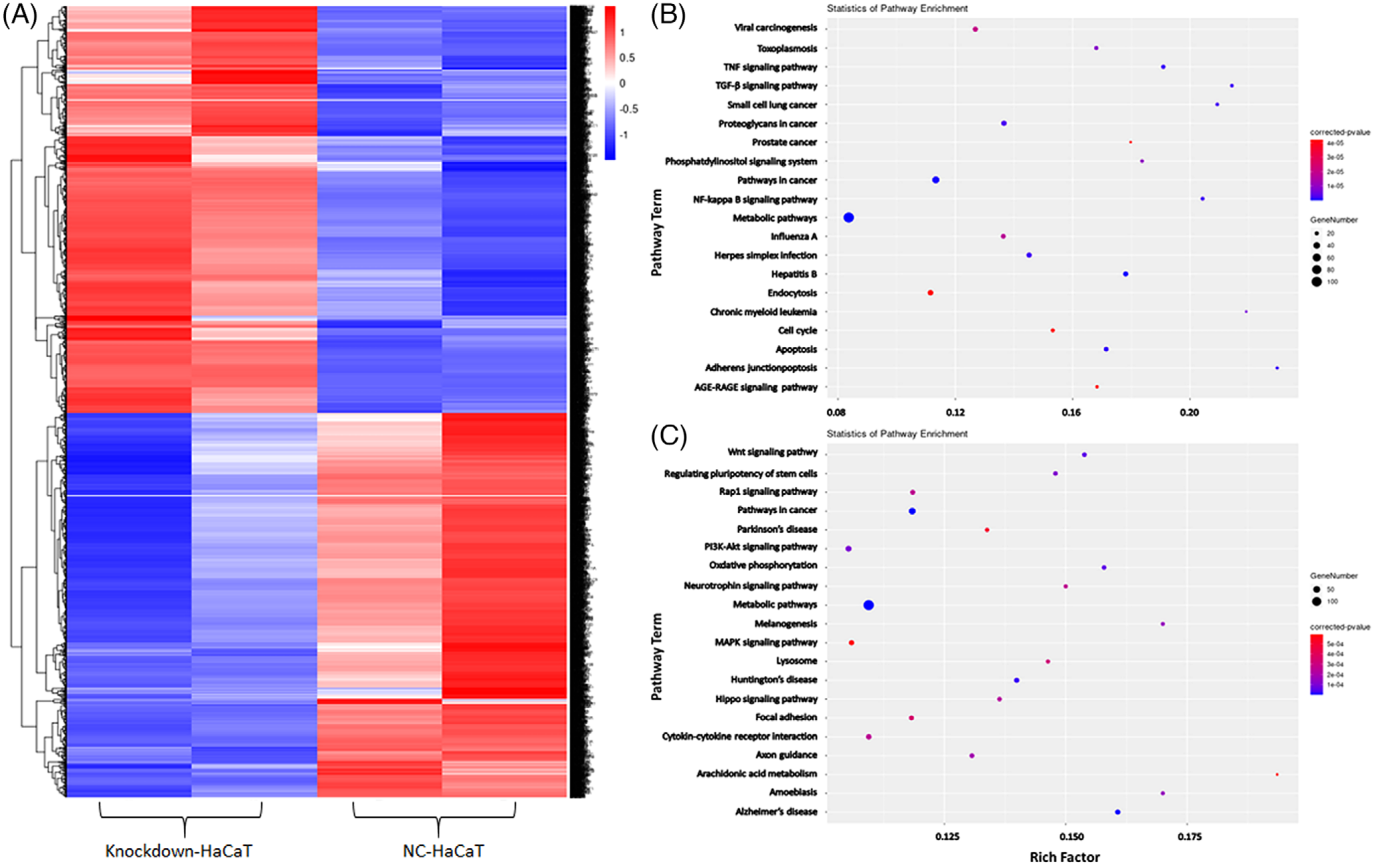
Livin expression could affect the genes in HaCaT cells. (A) Heat map of genes with significant differences of Livin knockdown‐HaCaT compared with NC‐HaCaT analysis by RNA‐seq (Different gene expression levels were indicated by different colors). (B) The KEGG pathway enrichment analysis revealed a close association of Livin with down‐regulated genes. (C) The KEGG pathway enrichment analysis revealed a close association of Livin with up‐regulated genes. KEGG, Kyoto Encyclopedia of Genes and Genomes.

Proteomic analysis and RNA sequencing were performed to examine the operational mechanisms of Livin. The heat map displays genes showing notable variations, with the sample represented on the abscissa and the filtered‐out differentially expressed genes represented on the ordinate. Expression levels ranging from low to high are represented by colors ranging from blue to white to red, where blue represents genes with low expression, and red represents genes with high expression (Figure 3A). Kyoto Encyclopedia of Genes and Genomes (KEGG) pathway enrichment analysis showed a strong correlation between the Livin and genes that were downregulated and upregulated in the inflammatory signaling pathway (Figure 3B,C).

**FIGURE 4:**
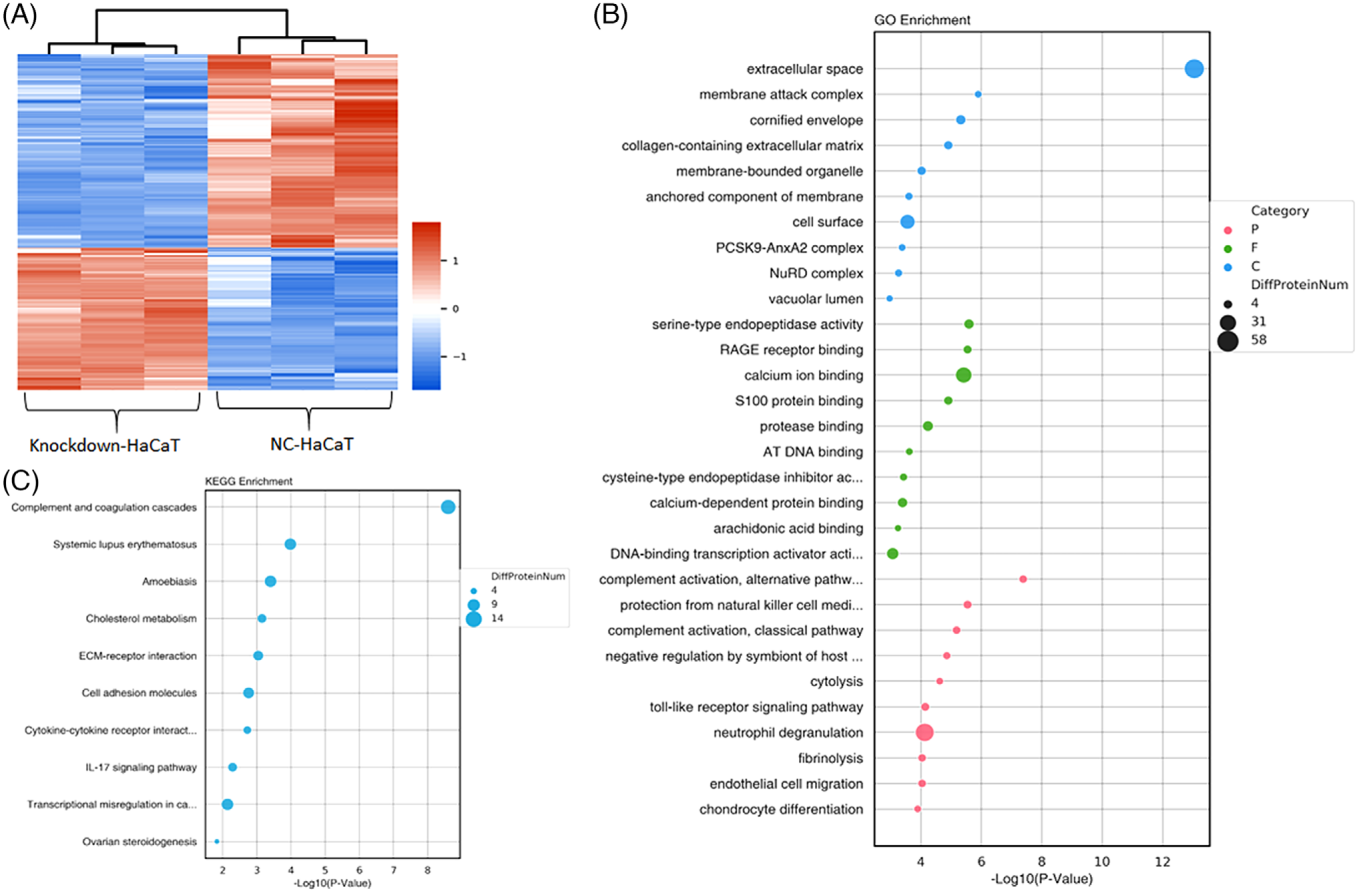
Livin expression could affect the proteins in HaCaT cells. (A) Heat map of genes with significant differences of Livin knockdown‐HaCaT compared with NC‐HaCaT analysis by proteomics (Different gene expression levels were indicated by different colors). (B–C) GO enrichment analysis and KEGG pathway enrichment analysis of Livin. GO, gene ontology; KEGG, Kyoto Encyclopedia of Genes and Genomes.

The heat map displays proteins with notable variances, with the sample represented on the abscissa and filtered out differentially expressed proteins on the ordinate. Different protein expression levels are indicated using different colors. For example, the expression levels from low to high are represented by colors ranging from blue to white to red, with blue indicating proteins that are at a low level and red indicating proteins expressed at a high level (Figure 4A). Livin function was elucidated using Gene Ontology (GO) and KEGG pathway enrichment analyses. GO enrichment analysis revealed a strong association between Livin in the knockdown group and the regulation of inflammatory responses, such as S100 protein binding and the Toll‐like receptor signaling pathway (Figure 4B). KEGG pathway analysis showed that Livin is closely linked to the complement and coagulation cascades, cytokine–receptor interactions, and IL‐17 signaling pathways, all of which play a significant role in psoriatic inflammation (Figure 4C).

**FIGURE 5:**
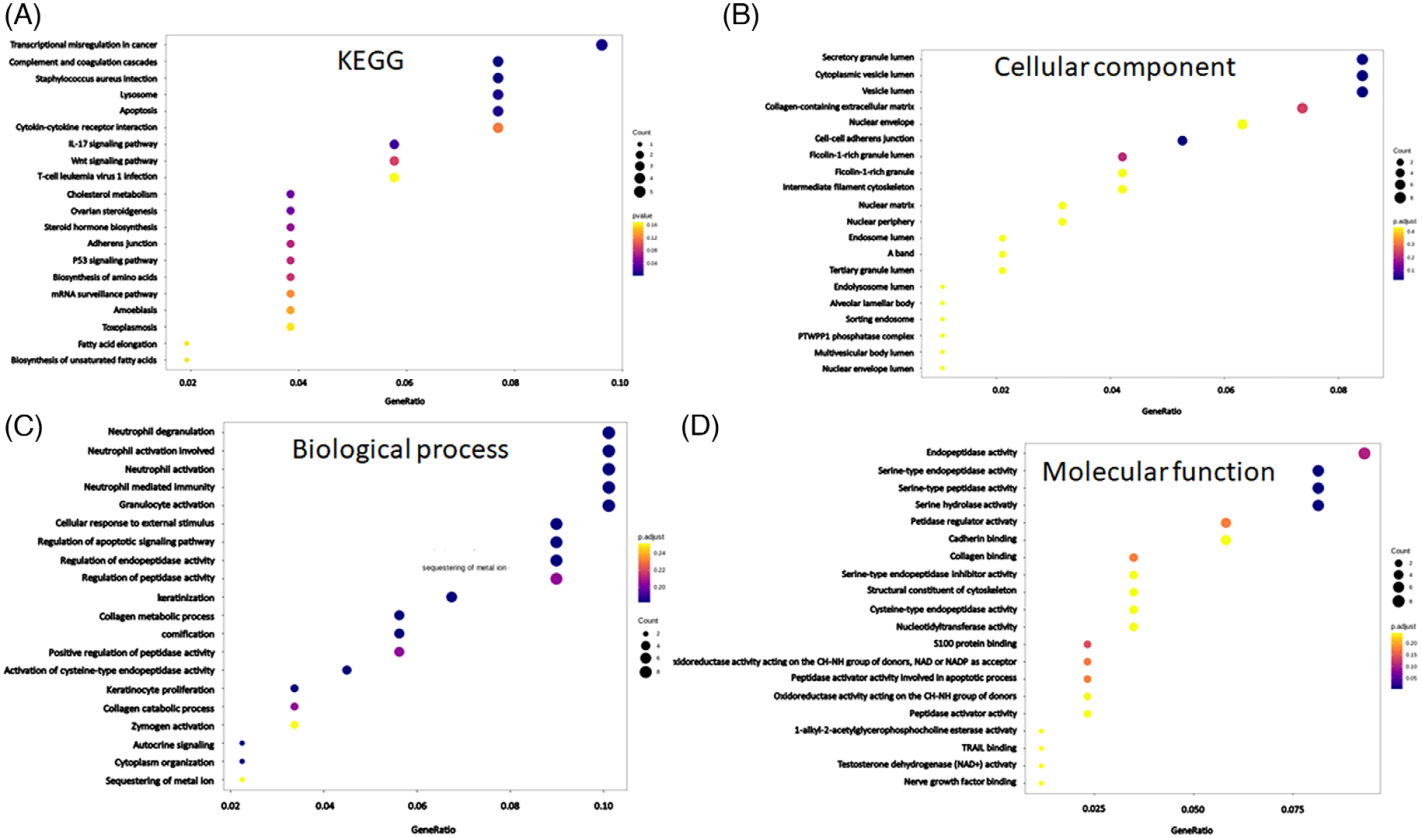
The intersection of proteomics and RNA‐seq of Livin. (A) The KEGG pathway enrichment analysis revealed a close association of Livin. (B–D) Livin were categorized based on CC, BP, and MF. BP, biological processes; CC, cellular components; KEGG, Kyoto Encyclopedia of Genes and Genomes; MF, molecular functions.

We further clarified the function of Livin using proteomic analysis and RNA sequencing. GO and KEGG pathway enrichment analyses were used to analyze genes and proteins expressed at lower levels. KEGG pathway analysis showed that livin was closely linked to the complement and coagulation cascades, interactions between cytokines and their receptors, and the IL‐17 signaling pathway (Figure 5A). Livins are classified according to their cellular components (CC), biological processes (BP), and molecular functions (MF). GO CC analysis showed that Livin levels were significantly enriched in the lumen of secretory granules, cytoplasmic vesicles, and vesicles (Figure 5B). In the BP group, the Livin levels were mainly enriched in regulating neutrophils participating in inflammatory responses and KC proliferation (Figure 5C). In the MF group, Livin showed enriched endopeptidase activity (Figure 5D).

### 3.3 Livin affects HaCaT cell activation

**FIGURE 6:**
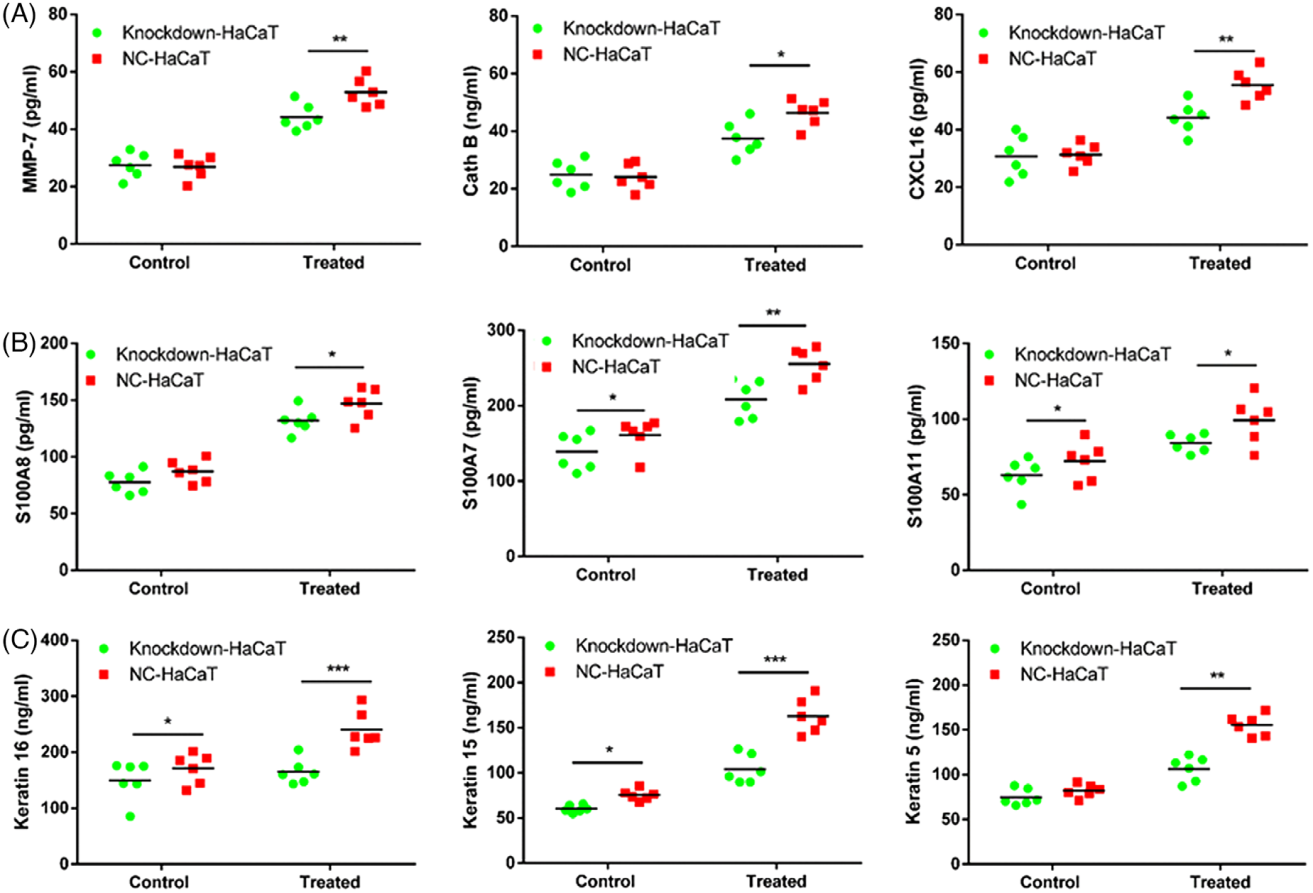
Livin expression showed effect on the inflammatory mediators release and activation in HaCaT. (A–B) MMP‐7, Cath B, CXCL16, S100A8, S100A7, and S100A11, were significantly decline after Livin expression down‐regulated in the control and treated groups. (C) Keratin16, keratin15, and keratin5 were significantly decline in the control and treated groups (data are expressed as mean ± standard error of the mean (SEM) and analyzed one‐tail paired Student's *t*‐test. Differences were considered significant at ^*^ *p* < 0.05, ^**^ *p* < 0.01, ^***^ *p* < 0.001).

To validate the impact of Livin on inflammatory agent release and triggering, the concentrations of matrix metalloproteinases (MMPs), peptides related to S100 protein, and keratin were assessed in M5‐treated and control groups. Following the decrease in Livin expression, a substantial reduction was observed in the levels of inflammatory mediators, namely, MMP‐7, Cath B, CXCL 6, S100A8, S100A7, and S100A11, which have crucial functions in psoriasis (Figure 6A). Moreover, the levels of keratins 16, 15, and 5, indicators of KC activation, were significantly downregulated (Figure 6B).

## 4 DISCUSSION

In addition to functioning as a mechanical barrier, KCs are important components of the innate immune system. KCs have crucial functions in initiating, maintaining, and controlling skin immune reactions and can trigger quick nonspecific responses to foreign substances and external triggers. During the early phase of psoriasis, KCs are activated by various stressors, such as injury, stimulation, and cytokines, resulting in increased proliferation and migration.

KCs produce a range of immune‐related proteins that contribute to psoriasis development. Another crucial role of KCs is the release of various antimicrobial peptides. Antimicrobial peptides are significantly elevated in psoriatic lesions, decreasing after effective treatment and acting as chemotactic immune cells regulating immune response.^10^, ^11^ Under the influence of external factors, such as infection and injury, KCs produce different antimicrobial peptides that regulate inflammation. However, the antimicrobial peptide LL‐37 can bind to the KC DNA or RNA to form a complex that activates Toll‐like receptors after endocytosis by plasmacytoid dendritic cells and mediates the production of TNF–α and IL‐23.^12^ Studies on KC‐conditioned genetically modified mice have revealed that KC dysfunction directly induces systemic autoimmune responses. Following the removal of two subunits from the transcription factor AP1, specifically JunB and c‐Jun, the mice exhibited a spontaneous resemblance to the chronic inflammatory characteristics observed in human psoriatic dermatitis and arthritis. Furthermore, the inflammatory response in mice does not depend on T‐cell activation.^13^ In mice, the excessive expression of the activated transcription factor STAT3 leads to the development of a severe psoriatic skin lesion phenotype.^14^ However, studies on endogenous regulatory factors involved in abnormal KC activation and proliferation are lacking.

Livin is a newly discovered anti‐apoptotic factor that inhibits apoptotic proteins. It is a 39‐kD protein encoded by a baculoviral IAP repeat‐containing seven genes located on Q13 of chromosome 20. Livin is abundantly expressed in undifferentiated tissues and controls cellular apoptosis, cell differentiation, and the cell cycle. Conversely, its expression is infrequent in terminally differentiated adult tissues.^15^, ^16^ Livin expression is observed in numerous tumor tissues, including malignant melanoma, renal cancer, and lung cancer.^17^, ^18^ Livin overexpression in tumor tissues may be associated with aggressive tumor behavior.

Livin suppresses apoptotic proteins, and its expression directly affects cancer cell growth and spread. Nevertheless, the role and activity of Livin in KCs and psoriasis remain unclear. Hence, Livin expression was determined in this study; Livin levels were significantly elevated in the skin lesions of patients with psoriasis and the IMQ mouse model. RNA sequencing and proteomic analyses were performed to examine the operational mechanism of Livin in KCs. Our findings indicate that Livin expression significantly affects KC activation, leading to the release of inflammatory mediators and upregulation of keratin. Livin controls KC activation and contributes to psoriasis‐related inflammation by releasing inflammatory mediators.

The BIR domain, located at the *N*‐terminus of Livin, plays a crucial role in preventing apoptosis. It specifically interacts with caspase‐3, 7, and 9 as well as other regulators of apoptosis, effectively suppressing their enzymatic activities and decreasing the population of malignant cells.^19^ However, whether abnormal KC proliferation is related to Livin expression in psoriasis has not been investigated. Our study confirmed that Livin was associated with abnormal KC activation in psoriasis. The increase in Livin expression resulted in elevated KC expression and stimulated the secretion of KC antimicrobial peptides and inflammatory factors. The activation of Toll‐like receptors in psoriasis triggers the release of antimicrobial peptides and inflammatory factors by activating the nuclear factor (NF)‐κB signaling pathway, leading to KC activation. The primary physiological function of Livin is the inhibition of caspase‐3 expression. The synthesis and release of KC inflammatory factors are directly linked to NF‐κB signaling pathway activation, potentially serving as a key mechanism for its involvement in regulating KC activation and the formation of inflammatory micro‐rings in psoriasis.^20^, ^21^ Livin suppressed caspase‐3 activity, and an inverse relationship was noted between their expression levels, thereby enhancing NF‐κB signaling pathway activation. The correlation between NF‐κB signaling pathway activation and the synthesis and release of inflammatory factors may serve as a key mechanism through which Livin mediates inflammatory microring formation in psoriasis.

## 5 CONCLUSIONS

To summarize, our study indicated that the Livin levels were elevated in the KCs of individuals with psoriasis. Livin expression affected KC activation, induced the release of inflammatory mediators, and upregulated keratin expression. This study presented a novel effector compound contributing to the mechanism of inflammatory reactions in psoriasis.

## CONFLICT OF INTEREST STATEMENT

The authors have no conflicts of interest to report.

## ETHICS STATEMENT

Human skin samples were conducted in strict accordance with the guidelines set by our institution and with the approval of the Ethics Committee of Xi'an Jiaotong University (2022‐1040). Animal experimental protocols were approved by Animal Ethics Committee at Xi'an Jiaotong University (Permit Number: XJTU 2021‐1517).

## Supporting information

Supporting Information

## Data Availability

The data that support the findings of this study are available upon request from the corresponding authors.
