# Supplementary material for: Livin expression promotes keratinocyte release of inflammatory mediators in psoriasis
Source: Skin Res Technol. 2024 Feb 8;30(2):e13603. doi: 10.1111/srt.13603 (PMC10853572; doi:10.1111/srt.13603)
Supplement: Supplementary file 1 — Supporting Information [file SRT-30-e13603-s001.docx]

**Supplementary figure**

**
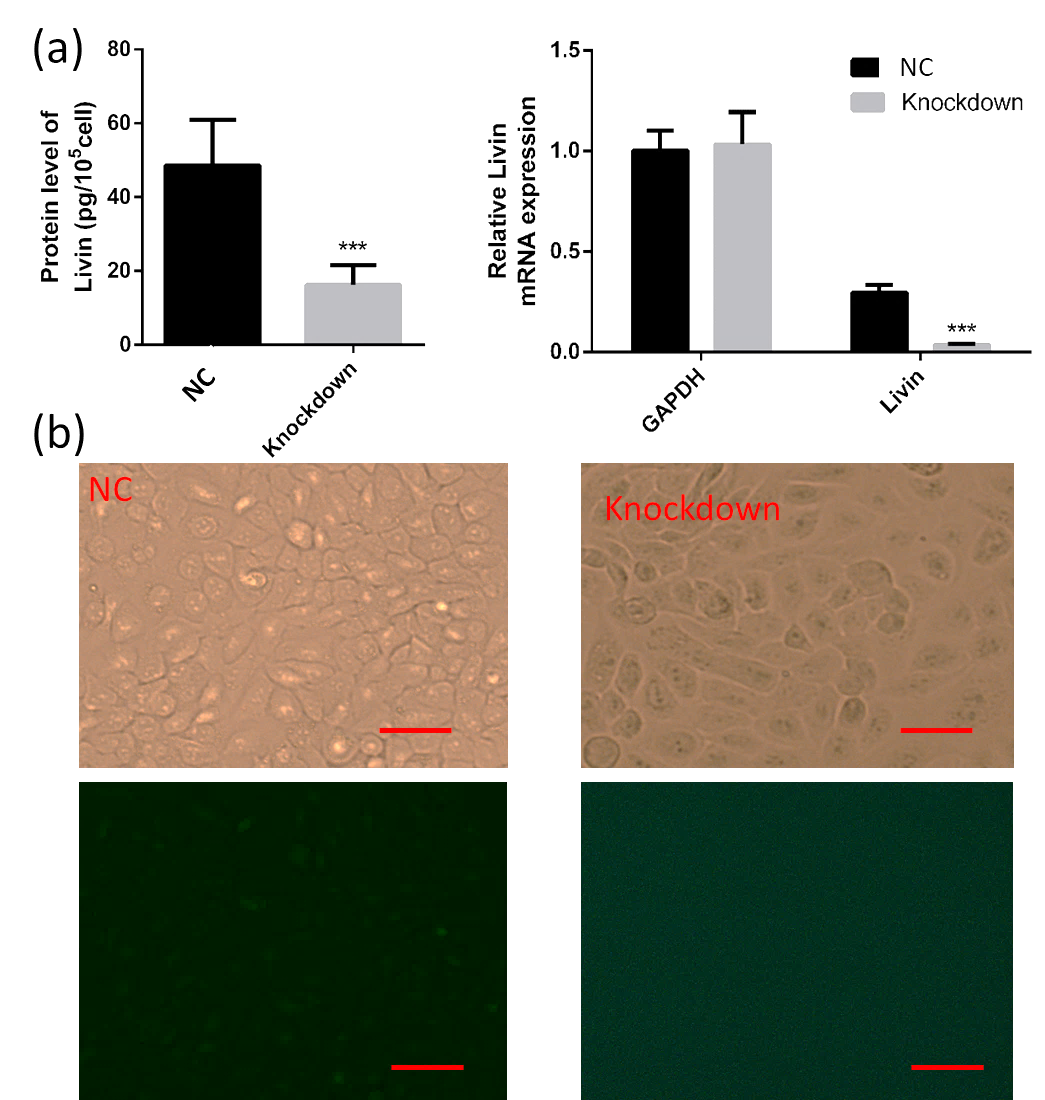
**

**Supplementary figure 1.** The expression of Livin in knockdown-HaCaT and negative NC-HaCaT cells. A: Livin expression analysis by ELISA and RT-qPCR. B: Livin expression analysis by immunofluorescent staining. (data are expressed as mean ± S.E.M. and analyzed one-tail paired Student’s t-test. Differences were considered significant at *** *p* < 0.001).
